# Quality of Life and Functional Impairment After Surgical Treatment of Pilon Fractures—A Case–Control Study with SF-12, EQ-5D-5L and VAS

**DOI:** 10.3390/jcm14196965

**Published:** 2025-10-01

**Authors:** Andreas Gather, Ann-Sophie C. Weigel, Benno Bullert, Axel Schumacher, Paul Alfred Gruetzner, Benedict Swartman

**Affiliations:** 1 Department of Interdisciplinary Emergency and Acute Medicine (IRN), BG Clinic Ludwigshafen, 67071 Ludwigshafen, Germany; 2 Department of Trauma, Hand and Reconstructive Surgery, University Hospital Münster, 48149 Münster, Germany; 3 Department of Trauma and Orthopaedic Surgery, BG Clinic Ludwigshafen, 67071 Ludwigshafen, Germany

**Keywords:** tibial fractures, fracture management, treatment outcome, quality of life, pain measurement

## Abstract

**Background:** Pilon fractures are severe distal tibia injuries from high-energy trauma, often involving joint and soft tissue damage. Despite surgical advances, long-term outcomes remain poor. This study compared quality of life and functional limitations after surgical treatment of pilon versus tibial shaft fractures using validated PROMs. **Methods:** This case–control study was conducted at a Level I Trauma Center. Between 2016 and 2019, 84 patients with lower leg fractures were included: 38 pilon and 46 tibial shaft fractures. Inclusion criteria were AO type 42 or 43 fractures and follow-up of ≥24 months; exclusion criteria were polytrauma (ISS > 15), ASA ≥ 3, and incomplete consent. Outcomes were assessed with SF-12, EQ-5D-5L, and VAS-FA. Data were collected 36–48 months postoperatively. Analyses included *t*-tests, chi-square tests, linear regression. **Results:** Patients with pilon fractures had significantly poorer physical quality of life than tibial shaft fractures (SF-12 physical: 39 vs. 42, *p* < 0.05). Mental quality of life showed no significant difference. EQ-5D-5L scores were lower in the pilon group (70% vs. 79%). VAS-FA indicated higher pain and reduced function (total: 64 vs. 76, *p* = 0.009). Rehabilitation duration correlated with improved VAS outcomes in pilon fractures (*p* = 0.008), while physiotherapy reduced pain in tibial shaft fractures (*p* = 0.030). **Conclusions:** Pilon fractures substantially impair physical quality of life and long-term function, while mental well-being remains unaffected. PROMs provide insights beyond radiological findings and should be integrated into follow-up. Further multicenter studies are required to validate these results and optimize rehabilitation strategies.

## 1. Introduction

Pilon fractures represent a particular challenge in trauma surgical care. As rare but highly complex injuries in the area of the distal tibia, they are typically the result of high-energy trauma and are associated with considerable structural destruction of the ankle joint and often also the distal fibula. Epidemiologically, they account for around 5–10% of all lower leg fractures and disproportionately affect men in younger and middle age groups, which can be attributed to occupational and sport-related accident mechanisms [1,2]. In recent years, an increasing number of cases have also been observed in older patients, often due to low-energy trauma in the context of osteoporosis, which underlines the heterogeneity of the patient population [3].

Biomechanically, these fractures usually involve axial forces acting on the talocrural joint, which can lead to complex joint fissures. The term ‘pilon’—introduced by Destot—refers to the French term for a pestle, which aptly describes the type of force application [4]. Due to the combination of high energy impact and complex anatomy of the distal tibia, concomitant soft tissue injuries are common, which further complicates surgical treatment and significantly influences the functional outcome [5,6,7,8,9]. Recent studies highlight that the extent of soft tissue damage is often a stronger predictor of complications than the actual fracture morphology [8].

Therapeutic strategies range from primary stabilization using an external fixator to definitive open reduction and plate osteosynthesis. The choice of procedure depends largely on the extent of the fracture, the soft tissue damage and the patient’s general condition. Despite technical advances, the functional outcome is often unsatisfactory. In particular, the postoperative quality of life and return to everyday life or work are often protracted and associated with permanent restrictions [10]. Two-stage procedures with initial external fixation followed by delayed definitive osteosynthesis have become increasingly established in the past years to reduce soft tissue complications [7]. Nevertheless, complication rates such as infection, pseudarthrosis or post-traumatic arthritis remain considerable, which continues to be confirmed by recent meta-analyses [11,12].

While radiological healing processes are routinely documented, the subjective experience of patients—in the form of pain, loss of mobility or psychological stress—remains underrepresented in many studies. Patient-Reported Outcome Measures (PROMs) such as the SF-12, the EQ-5D-5L or the Visual Analogue Scale for Foot and Ankle (VAS-FA) enable a differentiated assessment of these aspects [13]. Their use is becoming increasingly important, especially for complex fracture entities with a high functional risk. In the last three years, several publications have emphasized the role of PROMs as key indicators for long-term recovery and reintegration, complementing traditional clinical and radiological follow-up [3,9,14].

Despite international research literature, German-speaking countries have so far been underrepresented with regard to systematic surveys on quality of life after surgical treatment of pilon fractures. The few existing studies mostly focus on surgical techniques and radiological results, while comprehensive analyses of subjective health, functionality and restrictions relevant to everyday life are scarce [15]. Given the socioeconomic impact of prolonged incapacity for work and the high costs of complex inpatient care, further systematic investigation of patient-centered outcomes is warranted [3].

The aim of this study is therefore the targeted investigation of health-related quality of life and functional impairments in patients following surgical treatment of a pilon fracture. Validated survey instruments (SF-12, EQ-5D-5L, VAS) will be used to quantify both physical and psychological stress over the long-term. In addition, socio-demographic factors, type of therapy and complications will be analyzed for their impact on the subjective outcome.

## 2. Methodology

The present study was conducted as a case–control study at a level 1 trauma center. The aim was to record and evaluate the subjective quality of life and functional impairments following surgical treatment of pilon fractures. The survey was conducted at, a level 1 trauma center. While the retrospective part of the study served to identify suitable cases, standardized questionnaires were used to record the patients’ state of health over the long term. Consent was obtained from the ethics committee of the Rhineland-Palatinate Medical Association. All procedures complied with the Declaration of Helsinki and followed Good Clinical Practice (GCP) standards. The study was reported in accordance with STROBE recommendations for observational research.

The inclusion criteria included a minimum age of 18 years at the time of the accident, the presence of a distal tibia fracture (AO classification 42 and 43), including both extra-articular and intra-articular types. Patients were later sub grouped according to AO classification: 43 (intra-articular Pilon fractures) and 42 (extra-articular diaphyseal/metaphyseal fractures) and inpatient treatment in the period from 1 January 2016 to 31 December 2019. The minimum follow-up period was approximately 24 months for patients treated in 2019, with a maximum of 6 years for cases from 2016. No strict minimum follow-up interval was defined a priori; however, all included patients were contacted for outcome assessment between September 2021 and December 2022. Patients could be included regardless of whether they were undergoing initial, secondary or further treatment. The prerequisite for participation in the prospective part of the study was a signed written declaration of consent for the use of the collected data. Patients with proximal tibia fractures (AO 41), pure ankle fractures (AO 44), polytraumas with an Injury Severity Score (ISS) > 15 or an ASA score ≥ 3, as well as cases with two or more fractures or incomplete consent were excluded.

General data regarding number and duration of procedures, type of treatment, complications and incapacity of work were reported. Surgical treatment followed institutional standards for distal tibia fractures, including open reduction and internal fixation with locking plates or intramedullary nails. External fixation was applied in highly comminuted cases or in damage control situations. Perioperative antibiotic prophylaxis and thromboprophylaxis were administered according to national guidelines. Postoperative aftercare consisted of immobilization in a vacuum orthosis, followed by gradual weight-bearing after 6–8 weeks under physiotherapeutic supervision. Rehabilitation protocols were standardized and documented. Additional perioperative parameters such as hospital length of stay and the need for revision surgery were also documented to provide a more comprehensive description of the treatment course.

Three validated measurement instruments were used for the standardized recording of the subjective state of health. The Short Form-12 Health Survey (SF-12) was used to measure health-related quality of life in two separate scales for physical and mental health. The results were interpreted using established reference values [13]. In addition, the EQ-5D-5L was used, an internationally validated instrument for recording the general state of health in five dimensions: Mobility, self-care, coping with everyday life, pain and psychological well-being. In addition, the subjective assessment of the current state of health was recorded using a vertical visual analogue scale (VAS) from 0 (‘worst imaginable state of health’) to 100 (‘best imaginable state of health’) [16]. Finally, the VAS Foot/OSG Scale (VAS-FA), a differentiated questionnaire for recording pain, mobility and other complaints in the foot and ankle, was used for the specific assessment of foot-related functional limitations [17]. All questionnaires were used in their validated German versions to ensure comparability of results. Non-responders were documented, and sensitivity analyses excluding them were performed.

The data was collected by letter writing or by telephone. Patients received an information pack by letter containing questionnaires, a declaration of consent and a stamped envelope. If there was no response, the documents were sent out again. After a further twelve weeks, contact was made by telephone with up to ten contact attempts per person. Patients with distal tibia fractures (AO 42) without joint involvement, who were treated and interviewed under identical conditions, served as a control group. Both groups were tested not to have significant differences regarding age, gender and secondary diagnoses in order to minimize potential biases. All data were entered into a secure electronic database (REDCap^®^, Vanderbilt University, Nashville, Tennessee, USA) with access restricted to study staff. Double data entry was performed for accuracy. Regular monitoring was carried out by an independent investigator not involved in treatment.

The data was initially analyzed descriptively with mean values, standard deviations and frequencies. Group comparisons between pilon and tibia fractures were performed using the *t*-tests for independent samples or non-parametric methods (e.g., Mann–Whitney U test). Categorical data were analyzed using the Chi-squared test. Multiple linear regression analyses were carried out to investigate potential factors influencing quality of life. The significance level was set at *p* < 0.05. IBM SPSS Statistics^®^ Version 27 (IBM Corp., Armonk, NY, USA) software was used for the statistical analyses. Effect sizes (Cohen’s d, odds ratios) and 95% confidence intervals were reported for all major outcomes. To control for multiple testing, Bonferroni correction was applied where appropriate. Missing data were analyzed using multiple imputation (five iterations). Sensitivity analyses confirmed robustness of the results. Normal distribution was checked by Shapiro–Wilk test, variance homogeneity by Levene’s test. All statistical analyses were conducted using IBM SPSS Statistics^®^, Version 27 (IBM Corp., Armonk, NY, USA), and figures were prepared with GraphPad Prism^®^, Version 9 (Graphpad Software, Boston, MA, USA).

## 3. Results

**Figure 1:**
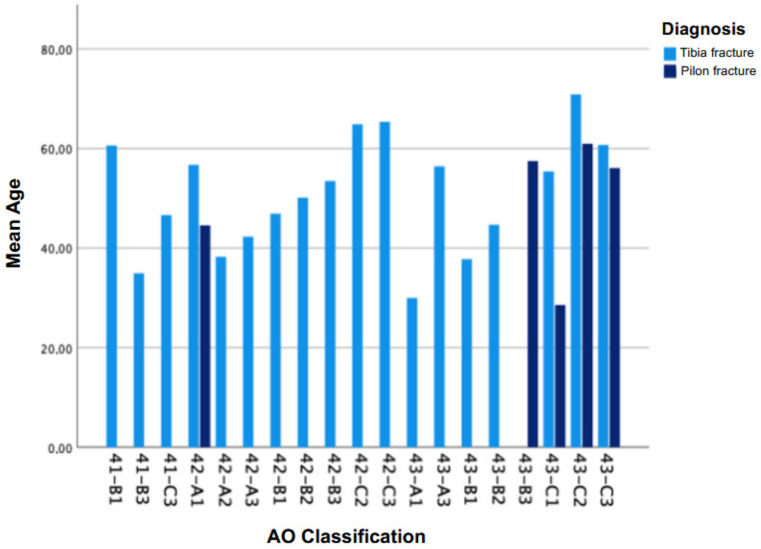
Mean age of patients with tibia and pilon fractures according to AO classification.

Between 2016 and 2019, a total of 183 patients with lower leg fractures were hospitalized at our institution. After applying the defined exclusion criteria, 157 patients were formally included and 84 patients could be reached. The sample consisted of 57 male (67.9%) and 27 female patients (32.1%). In the tibial fracture group (n = 46), 30 patients (65.2%) were male and 16 (34.8%) were female. In the pilon fracture group (n = 38), 27 patients (71.1%) were male and 11 (28.9%) were female. The difference in gender distribution between the groups was not statistically significant (*p* = 0.52, chi-square test) (see Table 1). The average age was 48 ± 14 years and the age range was 18 to 78 years. The age distribution was largely evenly distributed between 20 and 40 years, with a peak incidence between 55 and 60 years (see Figure 1). There was no significant age difference between the two fracture types (*p* > 0.05).

The ASA score (American Society of Anesthesiologists) was used to assess the general state of health. Only patients with an ASA score <3 were included in the study. In the total cohort, 79.8% were classified as ASA 1 and 20.2% as ASA 2. A subgroup analysis between tibial and pilon fracture patients was not performed for ASA classification. Therapeutic anticoagulation was not required for the majority of patients: 94% of the tibial fracture patients and 90% of the pilon fracture patients did not require long-term anticoagulation therapy.

The analysis of the insurance situation showed that the majority of patients had statutory health insurance. In the case of tibial shaft fractures, 50% were covered by statutory health insurance, while 45% of patients with pilon fractures were covered by statutory health insurance. The proportion of privately health insured patients in both groups was 13%. Patients insured by the employers’ liability insurance association accounted for 37% of tibial shaft fractures and 42% of pilon fractures.

### 3.1. Therapy and Surgical Parameters

The average number of operations was 1.7. In exceptional cases, a conservative approach was possible (n = 1; 1.2%). The average operation time was 149.40 min, whereby a maximum operation time of 360 min was documented for complex fractures with multiple plates. In pilon fractures, two-stage treatment using an external fixator was performed significantly more frequently (39.5% vs. 17.4% for tibia fractures). The most common treatment for tibial fractures was open reduction with plate osteosynthesis (39.1%), followed by closed reduction with plate osteosynthesis (30.4%) and nail osteosynthesis (26.1%). For pilon fractures, open reduction with plate osteosynthesis dominated (78.8%), followed by closed reduction and plate osteosynthesis (18.4%).

### 3.2. Complications and Soft Tissue Injuries

The overall rate of serious complications was comparatively low. In the tibial fracture cohort, 18 complications (39.1%) were documented, while 10 complications (26.3%) occurred in the pilon fractures. The osseous complications included pseudarthrosis and secondary dislocations. Two cases of pseudarthrosis occurred in the tibial fracture group (4.35%), while three cases (7.89%) were documented in the pilon fracture group. Soft tissue injuries were categorized according to the Tscherne-Oestern classification. Open fractures occurred in 11 cases in each group, with no statistically significant differences in Gustilo severity grades. Among closed fractures, grade 0 soft tissue injury was most common. However, high-grade soft tissue injuries (Tscherne grade III), including compartment syndromes, were more frequently observed in the tibial fracture group (19.6%, n = 9) compared to the pilon fracture group (7.9%, n = 3). Wound healing disorders occurred in one case of a pilon fracture (2.63%), while five cases (10.87%) were documented in tibial fracture group, including wound infections (6.52%) and complication wounds (2.17%). A nerve lesion of the saphenous nerve was found in one case in both groups.

### 3.3. Quality of Life and Functional Outcomes

The analysis of the physical state in the SF-12 revealed an average value of 42.8 ± 5.8 for patients with tibial fractures, while patients with pilon fractures achieved a lower value of 39.4 ± 6.1 (Table 1). An evaluation using regression analysis showed a significant correlation with the duration of rehabilitation and the duration of physiotherapy for tibial fractures (Table 2).

In comparison to the physical state of health, the mental state of health measured using the SF-12 (Mental State) showed no significant difference between the two patient groups. The tibia fracture patients scored an average of 43.6 ± 8.85, while the pilon fracture patients scored 45.4 ± 7.5 (Table 1). No significant correlation was found regarding to the influencing factors either (Table 3).

The EQ-5D-5L analysis showed that patients with tibial fractures reported an average score of 79%, while patients with pilon fractures only achieved 66% (Table 1). The regression analysis showed that a better subjective state of health was associated with a lower number of physiotherapy appointments for patients with tibial fractures (*p* = 0.009). In contrast, the factors rehabilitation duration and lymphatic drainage showed no significant effects. In the case of pilon fractures, a longer rehabilitation period was associated with a poorer state of health (*p* = 0.015) (Table 4).

The VAS values were collected on average 36 to 48 months after the accident. Several dimensions were taken into account: Pain, function, other (e.g., gait pattern, mobility) as well as an overall assessment (Table 1). A statistically significant correlation was found between the operation time and the total VAS foot/ankle score, with a longer operation time being associated with a lower VAS score on average. This correlation was also found for the duration of incapacity for work and rehabilitation time. This correlation between longer operation times and extended periods of incapacity for work and rehabilitation was observed across the overall study population. Further subgroup analysis is needed to determine whether this effect applies consistently to both tibial and pilon fractures. For patients with tibial fractures, the duration of physiotherapy was found to be statistically significant (α = 0.030) with a negative regression coefficient of −0.149, meaning that longer physiotherapy correlated with a lower VAS score. For pilon fractures, the duration of work incapacity was almost significant (α = 0.054) and also had a negative regression coefficient of −0.187, indicating a lower VAS score with longer work incapacity. In addition, the duration of rehabilitation for pilon fractures showed a significant influence (α = 0.008), whereby a longer rehabilitation period was associated with a lower VAS score, indicating less perceived pain at the time of follow-up.

In both the tibial and pilon fracture groups, a correlation was found between the final result of the functional test and the subjectively perceived state of health. The total VAS correlated with the ‘Health Today’ of the EQ-5D-5L with r = 0.736 for tibia fractures and with r = 0.725 for pilon fractures. This correlation is more pronounced than that of the SF-12 both in the physical condition (tibia fractures: r = 0.520; pilon fractures: r = 0.416) and in the mental condition (tibia fractures: r = 0.480; pilon fractures: r = 0.424).

### 3.4. Incapacity for Work and Rehabilitation

The average duration of incapacity for work was 31 weeks for tibia fractures and 42 weeks for pilon fractures. The duration of rehabilitation was significantly longer for pilon fractures, with a mean of 17.8 weeks compared to 9.6 weeks for tibial fractures (*p* < 0.001), corresponding to an average difference of 8.2 weeks.

## 4. Discussion

This study investigated the quality of life and functional limitations following surgical treatment of pilon and tibia fractures. A total of 84 patients were recorded as case–control study, including 38 patients with pilon fractures and 46 patients with tibia fractures. The main results show that the physical quality of life (SF-12) is significantly worse for patients with pilon fractures than in patients with tibia fractures, while the mental quality of life remains comparable. The subjective perception of health (EQ-5D-5L) was also worse for patients with pilon fractures. The pain assessment using VAS showed a higher pain intensity and limited functionality for pilon fractures compared to tibia fractures. A longer rehabilitation period for pilon fractures was significantly associated with lower VAS scores, indicating less perceived pain at follow-up. This may reflect the benefit of structured, longer-term inpatient rehabilitation, especially after complex intra-articular injuries. For tibial fractures, a longer duration of outpatient physiotherapy also correlated with improved pain outcomes. These findings highlight the relevance of sustained rehabilitative care across both groups. Additionally, incapacity to work was significantly longer for pilon fractures, likely reflecting the higher complexity, longer healing times, and slower functional recovery compared to extra-articular tibial fractures.

Interestingly, a statistically significant negative correlation was observed between operation time and the total VAS foot/ankle score. This suggests that longer surgical procedures were associated with worse subjective outcomes regarding pain and function. One possible explanation could be the increased soft tissue manipulation and complexity in longer surgeries, which may negatively impact postoperative recovery and rehabilitation. This finding highlights the importance of efficient surgical planning and timing, especially in complex fracture cases. Future studies should further evaluate whether surgical duration serves as an independent predictor of functional outcome or reflects the underlying complexity of the injury.

The poorer physical quality of life observed for pilon fractures in this study is consistent with the results of Pollak et al. who also described a significant reduction in physical function after complex fractures [18]. Lewis et al. also reported that mental quality of life is less impaired, which is consistent with the present results [19].

With regard to subjective health perception (EQ-5D-5L), the results show parallels to the study by Bonato et al., which documented a reduced quality of life, particularly for patients with complex ankle fractures [20]. These authors also found that patients with pilon fractures reported long-term persistent limitations, particularly in the area of physical mobility.

Pain assessment using VAS showed that the pilon fracture group reported higher pain intensity than the tibia fracture group. This is consistent with the findings of Renzi Brivio et al. who also documented a persistent pain burden after complex fractures [21].

It is important to note that the terms “physiotherapy duration” and “rehabilitation duration” refer to different aspects of postoperative care in this study. For tibial fractures, the duration of physiotherapy—typically referring to the number or length of outpatient physiotherapy sessions—showed a significant negative association with VAS scores. This suggests that longer physiotherapeutic support is beneficial for pain reduction and functional recovery. In contrast, for pilon fractures, a longer duration of inpatient rehabilitation was associated with lower VAS scores as well, although the correlation with incapacity for work was only marginally significant. However, this finding may reflect a reversed causality: patients with more complex injuries or complications likely required prolonged inpatient rehabilitation, which in turn indicates a more severe initial clinical condition. Thus, the length of rehabilitation in this context may act as a surrogate marker for injury severity rather than a direct cause of lower functional scores.

Another important point is the prolonged incapacity to work for patients with pilon fractures. Similar results were reported by Levy et al. who found that complex ankle fractures often lead to prolonged sick leave [22]. The present study also showed that prolonged incapacity for work for pilon fractures correlates with poorer VAS scores, which is also consistent with the findings of Wu et al. who described the negative influence of prolonged convalescence times on functional recovery [23]. The positive effect of physiotherapy on tibial fractures, which was significantly demonstrated in this study, should be emphasized in particular.

The present results confirm the functional deficits described in the literature and the reduced quality of life for patients with pilon fractures. The significantly poorer physical quality of life and the greater pain burden emphasis the need for appropriate aftercare, particularly in the case of complex injuries. Interestingly, longer inpatient rehabilitation in patients with pilon fractures was associated with lower VAS scores at follow-up, indicating a beneficial effect on pain. This may reflect the positive impact of comprehensive, multidisciplinary rehabilitation programs that are more frequently applied in cases of extended stays. Rather than indicating greater injury severity, longer rehabilitation in this context may act as a proxy for structured and sustained care, which ultimately contributes to improved functional outcomes.

The prolonged incapacity to work in the case of pilon fractures indicates the need to customize the return to work and to plan long-term rehabilitation measures. Greater integration of physiotherapy concepts in complex ankle injuries could minimize functional deficits and facilitate reintegration into the workplace.

Future studies should be multicenter in order to validate the results and specifically investigate the factors influencing better functional recovery. The long-term consequences of pilon fractures in particular deserve further attention in order to be able to better customize individual treatment approaches.

The present study also showed that a longer incapacity for work for pilon fractures correlates with poorer VAS values, which is also consistent with the findings of Wu et al. who described the negative influence of prolonged convalescence times on functional recovery.

## 5. Conclusions

The present study shows that patients with pilon fractures have a significantly poorer physical quality of life and a higher pain burden in the long term compared to tibia fractures. While mental quality of life is largely comparable, functional limitations and prolonged incapacity to work are particularly pronounced for pilon fractures. Pilon fractures have a lasting impact on quality of life, particularly in the physical area, which requires a targeted adaptation of rehabilitation program in order to specifically promote functionality. Nevertheless it has to be mentioned that prolonged rehabilitation period negatively influenced subjective pain measures after pilon fractures.

In summary, pilon fractures are associated with a markedly more complex treatment course, a higher frequency of staged surgical procedures and longer rehabilitation demands, underlining their clinical and socioeconomic relevance.

Certain limitations of this study should be acknowledged. The retrospective design and single-center setting may restrict generalizability. The moderate sample size and incomplete follow-up with non-responders may have introduced selection bias. Furthermore, subgroup analyses for comorbidities and ASA classification were limited by available case numbers, and no randomization or matched-pair analysis was performed.

Future research should aim at prospective, multicenter studies with larger cohorts and longer follow-up intervals to validate these findings. The integration of objective functional outcome measures, such as gait analysis and strength testing, alongside patient-reported outcomes is recommended. In addition, the development of standardized treatment and rehabilitation protocols for pilon fractures could contribute to reducing complication rates and improving long-term quality of life.

## Figures and Tables

**Table 1:** Overview of SF-12, VAS Foot/Ankle, and EQ-5D-5L in Tibial and Pilon Fractures.

Test	Tibial Fracture	Pilon Fracture
SF-12 physical (mean ± SD)	42.82 ± 5.77	39.36 ± 6.12
SF-12 mental (mean ± SD)	43.62 ± 8.85	45.41 ± 7.51
VAS pain (mean ± SD)	76.03 ± 22.90	66.08 ± 28.67
VAS function (mean ± SD)	73.97 ± 25.13	58.54 ± 26.92
VAS other (mean ± SD)	76.43 ± 21.74	64.18 ± 26.20
VAS total (mean ± SD)	76.68 ± 22.13	64.26 ± 25.02
EQ-5D-5L Health Today (mean ± SD)	79.7 ± 17.72	66.05 ± 25.25

**Table 2:** Multiple regression with physical component of SF-12 as the dependent variable.

Diagnosis	Variable	B	Std.-Error	t	Sig.	Lower Bound	Upper Bound
Tibial fracture	Operation time (min))	−0.014	0.011	−1.259	0.216	−0.037	0.009
Tibial fracture	Work incapacity (weeks)	−0.054	0.037	−1.467	0.150	−0.128	0.020
Tibial fracture	Rehabilitation (weeks)	0.250	0.107	2.342	0.024	0.043	0.466
Tibial fracture	Physiotherapy (weeks)	−0.035	0.017	−2.000	0.053	−0.070	0.000
Tibial fracture	Lymphatic drainage (weeks)	−0.18	0.019	−0.993	0.327	−0.056	0.019
Pilon fracture	Operation time (min))	−0.010	0.021	0.497	0.622	−0.032	0.053
Pilon fracture	Work incapacity (weeks)	−0.025	0.028	−0.874	0.389	−0.082	0.033
Pilon fracture	Rehabilitation (weeks)	−0.076	0.140	−0.546	0.589	−0.362	0.209
Pilon fracture	Physiotherapy (weeks)	−0.005	0.030	−0.185	0.855	−0.066	0.055
Pilon fracture	Lymphatic drainage (weeks)	−0.011	0.035	−0.332	0.742	−0.082	0.059

**Table 3:** Multiple regression with mental component of SF-12 as the dependent variable.

Diagnosis	Variable	B	Std.-Error	t	Sig.	Lower Bound	Upper Bound
Tibial fracture	Operation time (min)	−0.009	0.020	−0.441	0.661	−0.049	0.031
Tibial fracture	Work incapacity (weeks)	0.002	0.065	0.035	0.972	−0.129	0.134
Tibial fracture	Rehabilitation (weeks)	0.045	0.189	0.236	0.815	−0.338	0.427
Tibial fracture	Physiotherapy (weeks)	−0.009	0.031	−0.305	0.762	−0.072	0.053
Tibial fracture	Lymphatic drainage (weeks)	0.014	0.033	0.417	0.679	−0.053	0.080
Pilon fracture	Operation time (min)	−0.012	0.026	−0.465	0.645	−0.064	0.040
Pilon fracture	Work incapacity (weeks)	−0.012	0.034	−0.362	0.720	−0.083	0.058
Pilon fracture	Rehabilitation (weeks)	−0.067	0.170	−0.396	0.695	−0.415	0.280
Pilon fracture	Physiotherapy (weeks)	0.056	0.036	1.546	0.132	−0.018	0.129
Pilon fracture	Lymphatic drainage (weeks)	−0.030	0.042	−0.713	0.481	−0.116	0.056

**Table 4:** Multiple regression with “Health Today” (%) of EQ-5D-5L as the dependent variable.

Diagnosis	Variable	B	Std.-Error	t	Sig.	Lower Bound	Upper Bound
Tibial fracture	Operation time (min)	−0.051	0.035	−1.475	0.148	−0.122	0.019
Tibial fracture	Work incapacity (weeks)	−0.039	0.115	−0.339	0.737	−0.271	0.193
Tibial fracture	Rehabilitation (weeks)	0.545	0.337	1.614	0.114	−0.137	1.226
Tibial fracture	Physiotherapy (weeks)	−0.151	0.055	−2.733	0.009	−0.263	−0.039
Tibial fracture	Lymphatic drainage (weeks)	0.023	0.058	0.399	0.692	−0.095	0.142
Pilon fracture	Operation time (min)	−0.052	0.078	−0.673	0.506	−0.211	0.106
Pilon fracture	Work incapacity (weeks)	−0.100	0.102	−0.977	0.336	−0.309	0.109
Pilon fracture	Rehabilitation (weeks)	−1.330	0.516	−2.577	0.015	−2.382	−0.279
Pilon fracture	Physiotherapy (weeks)	0.080	0.110	0.730	0.471	−0.143	0.304
Pilon fracture	Lymphatic drainage (weeks)	−0.033	0.123	−0.267	0.791	−0.283	0.218

## Data Availability

The data presented in this study are available on reasonable request from the corresponding author. The data are not publicly available due to privacy restrictions.

## Abbreviations

AO	Arbeitsgemeinschaft für Osteosynthesefragen (fracture classification)
ASA	American Society of Anesthesiologists (physical status classification)
CI	Confidence Interval
EQ-5D-5L	EuroQol Five-Dimension Five-Level Questionnaire
GCP	Good Clinical Practice
IRB	Institutional Review Board
ISS	Injury Severity Score
PROMs	Patient-Reported Outcome Measures
SF-12	12-Item Short Form Health Survey
SPSS	Statistical Package for the Social Sciences
STROBE	Strengthening the Reporting of Observational Studies in Epidemiology
VAS	Visual Analogue Scale
VAS-FA	Visual Analogue Scale—Foot and Ankle
